# Research on GDP Forecast Analysis Combining BP Neural Network and ARIMA Model

**DOI:** 10.1155/2021/1026978

**Published:** 2021-11-12

**Authors:** Shaobo Lu

**Affiliations:** Cardiff University Business School, Cardiff CF10 3AT, Wales, UK

## Abstract

Based on the BP neural network and the ARIMA model, this paper predicts the nonlinear residual of GDP and adds the predicted values of the two models to obtain the final predicted value of the model. First, the focus is on the ARMA model in the univariate time series. However, in real life, forecasts are often affected by many factors, so the following introduces the ARIMAX model in the multivariate time series. In the prediction process, the network structure and various parameters of the neural network are not given in a systematic way, so the operation of the neural network is affected by many factors. Each forecasting method has its scope of application and also has its own weaknesses caused by the characteristics of its own model. Secondly, this paper proposes an effective combination method according to the GDP characteristics and builds an improved algorithm BP neural network price prediction model, the research on the combination of GDP prediction model is currently mostly focused on the weighted form, and this article proposes another combination, namely, error correction. According to the price characteristics, we determine the appropriate number of hidden layer nodes and build a BP neural network price prediction model based on the improved algorithm. Validation of examples shows that the error-corrected GDP forecast model is also better than the weighted GDP forecast model, which shows that error correction is also a better combination of forecasting methods. The forecast results of BP neural network have lower errors and monthly prices. The relative error of prediction is about 2.5%. Through comparison with the prediction results of the ARIMA model, in the daily price prediction, the relative error of the BP neural network prediction is 1.5%, which is lower than the relative error of the ARIMA model of 2%.

## 1. Introduction

GDP refers to the market value of all products and services produced by a country or region in a certain period of time using production factors. GDP not only is the accuracy of demand forecasting, but also provides a reference and basis for countries and regions in the deployment of strategic guidelines and the formulation of macroeconomic policies [1]. In addition, the statistics of GDP are more accurate and the calculation repeatability is small, so statistics are relatively easy [2]. GDP and economic growth rate, inflation rate, and unemployment rate are the main macroeconomic operation indicators that are closely related and are the most basic indicators [3]. In order to meet the requirements of social development and meet the needs of more accurate predictions, some prediction problems have introduced computer technology, mathematical methods, and logical reasoning. The gradual development and maturity of these systems have led many countries in the world to establish various predictions. Research departments provide decision-making references for economic development, provide strength support for strategic research, and guide enterprises and the country's current and future social activities to maximize utility [4–6].

In GDP forecasting, a single model is used for forecasting. When generating forecast results, we often discard the forecast errors. We think that the useful information has been extracted. In fact, the error contains a small part of the information, and it is still desirable [7–9]. Therefore, some scholars integrate existing prediction methods and use the promotion and complementation of different information to improve the prediction accuracy of the model. Many scholars have added error correction models when making GDP predictions [10]; the results show that the addition of the error correction model can significantly improve the accuracy of GDP prediction. Gao [11] et al. used a combination of ARIMA model and neural network in the forecast of tourism demand. Qin [12] et al. used SARIMABP to construct seasonal error correction time prediction model. In their article on carbon emissions prediction, they divided the data into linear and nonlinear parts, making full use of the good advantages of time series linear forecasting to make preliminary predictions, and then through the neural network implements error correction, the results show that the participation of the neural network is much better than the prediction effect of ARIMA alone. Yang [13] first used the adaptive filtering method to complete the preliminary prediction on the prediction data and then used Markov chain to correct the residuals generated after the preliminary prediction, and the result shows the feasibility of the method. Wang [14] further discussed the variable weight GDP forecast model and gave corresponding theoretical proofs and practical applications. Wang [15] proposed a generalized recursive reciprocal variance GDP prediction method on the basis of recursive equal weight GDP prediction and recursive reciprocal variance GDP prediction and deduced the iterative formula, starting from the GDP prediction accuracy. A combination model with the objective function as the predictive effectiveness index is established, and the optimal approximate solution of the weights is obtained. Some scholars comprehensively consider the mathematical expectation and standard deviation of GDP forecast accuracy, establish a multiobjective optimization GDP forecast model, derive its mathematical programming solution, and introduce entropy theory, and according to the degree of variation of the error sequence of the single forecast model, we obtain the weighting coefficient of each single model [16–19].

This paper studies and analyzes the artificial neural network model, especially the operating mechanism of BP neural network, compares its advantages with traditional forecasting methods, constructs a GDP forecast model, and discusses the setting rules of various parameters in the model. Experiments analyze the feasibility and superiority of artificial neural network in the field of GDP prediction and compare the prediction accuracy of BP neural network and traditional time series forecasting method. In the selection of prediction methods, the comparison is based on the superiority of the algorithm's BP neural network and the prediction accuracy of the BP neural network and the ARIMA prediction model. In order to reduce the prediction error and improve the accuracy of the model, this paper proposes a combination prediction model and a combination coefficient method to find a suitable model based on the information characteristics of the sequence value itself. From the final fitting results, it can be seen that the predicted value and the true value are very consistent; the true value falls within the 95% confidence interval, which fully shows that the model combination effect is good and the prediction accuracy is high. This empirical study shows that the ARIMA model and the ARIMAX model can be applied in actual work to make short-term macroforecasts.

## 2. Construction of the GDP Prediction Model Based on the BP Neural Network and ARIMA Model

### 2.1. BP Neural Network Spatial Sequence

**Figure 1:**
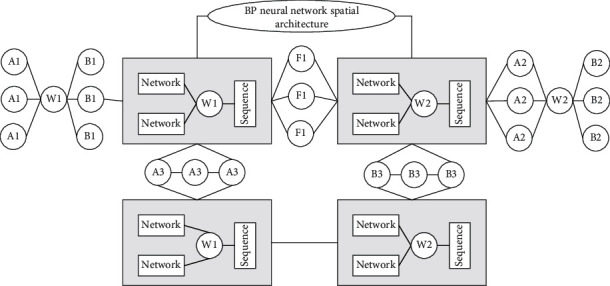
BP neural network spatial architecture.

The BP neural network is a computer-based processing system created by imitating the human brain. It is trained to understand the operating rules of the real system [20].  Figure 1 shows the spatial architecture of the BP neural network. Its outstanding ability is reflected in the prediction of nonlinear time series. It has strong predictive ability for exponentially increasing trend models, high accuracy, and strong fault tolerance, and fast information processing speed and can process quantitative and qualitative information at the same time, without the need to consider the system for problem of decoupling. The disadvantage is that the learning speed is very slow, the network training is very likely to fail, and it is more difficult to find the global minimum [21–24].

The establishment of the dynamic regression model is based on the assumption that there is a long-term equilibrium relationship between the response sequence and the independent variable sequence; that is to say, not all sequences can establish a dynamic regression model, only those sequences that have a long-term equilibrium relationship. It is suitable for establishing dynamic regression model [25, 26].

(1) $$\begin{array}{c} f\left(x|n\right)=\left\{x\left(1\right),x\left(2\right),\ldots ,x\left(n\right)|n\in R\right\}, \end{array}$$

(2) $$\begin{array}{c} Y\left(x\right)=\sum_{x=1,y=1}^{n}p\left(y|x\right)\times f\left(y|x\right). \end{array}$$

According to the introduction of the one-time exponential smoothing model, it is suitable for stationary series without changing trend. In order to apply to the time series with obvious upward or downward trend, a double exponential smoothing model is proposed. The double exponential smoothing model uses the first exponential smoothing model to process the data after the first exponential smoothing model for the second time [27, 28].

(3) $$\begin{array}{c} \left|x\left(1\right)-f\left(x\right)\right|+\left|x\left(2\right)-f\left(x\right)\right|+\cdots +\left|x\left(n\right)-f\left(x\right)\right|=n\times f\left(x\right), \end{array}$$

(4) $$\begin{array}{c} \frac{g\left(x\right)-\sum_{i,j=1}^{n}\left[s\left(1,i\right)+s\left(2,i\right)+\cdots +s\left(j,i\right)\right]}{s\left(i,j\right)}=0. \end{array}$$

Since random fluctuations are not actually measurable, when we actually make a model, we usually set the value of the unobserved value to 0 and then perform the least squares. This estimation is called because of the addition of the preconditions. When we are building a model, it is impossible to try all models. The first thing we have to do is to determine the selected model form and then determine the order of the model. Generally for this step, we usually call it the model order. It can be determined based on the ACF diagram and the PACF diagram.

(5) $$\begin{array}{c} \sigma \left(x,y\right)=\frac{1}{E}\times \left(\varepsilon \left(x,x\right)-t\left(\varepsilon \left(y,y\right)\right)\right), \end{array}$$

(6) $$\begin{array}{c} \left\{\sigma \left(x,x\right),\sigma \left(y,y\right),\sigma \left(z,z\right)\right\}⟶\left\{\varepsilon \left(x,y\right),\varepsilon \left(y,z\right),\varepsilon \left(z,x\right)\right\}. \end{array}$$

With the observation sequence, we have to study the properties of these sequences, but the properties of random sequences cannot be obtained intuitively. In this way, we indirectly study the properties of his observation sequence and then infer randomness through the properties of the observations. When a group of data containing gray information is obtained, this group of data is usually irregular, but there is always some kind of generation to process the data to make it regular.

(7) $$\begin{array}{c} U=\frac{1}{2}\times \int_{\Omega }\sigma \left(z,x\right)\sigma \left(x,y\right)\sigma \left(y,z\right)d\Omega . \end{array}$$

Based on this feature, we can process the data to build an equation generation model to achieve the fundamental mode of predicting the future. In general, the model constructed by the gray model is similar to a differential equation.

### 2.2. ARIMA Algorithm Structure Distribution

The Auto Regressive Integrated Moving Average model is abbreviated as the ARIMA (*p*, *d*, *q*) model, where *p*, *d*, and *q* represent the order of the autoregressive model, the order of the difference, and the order of the moving average model, respectively; ARIMA(*p*, *d*, *q*) model is essentially a combination of difference operation and autoregressive moving average. First, the sequence is subjected to *d* difference processing, transforms it into a stationary time series, determines the values of *p* and *q* according to the obtained stationary time series, and then uses the ARMA(*p*, *q*) model to predict and analyze the stationary time series. The aforementioned AR(*p*) model, MA(*q*) model, and ARMA(*p*, *q*) model are all stationary series. For nonstationary series, it is usually processed by difference processing several times first to convert them into stationary series. AR(*p*), MA(*q*), and ARMA(*p, q*) models are all established on the assumption that the time series are stationary.  Table 1 shows the composition of the time series factors.

**Figure 2:**
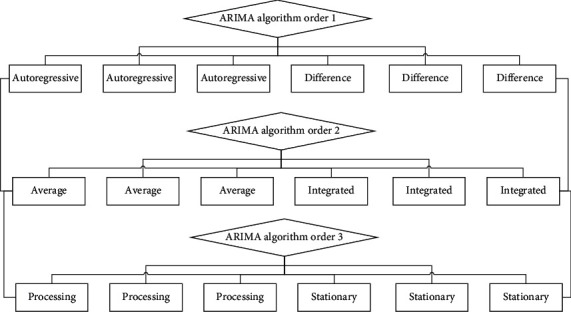
ARIMA algorithm structure topology.

There are a large number of nonstationary series in practical problems, so it is necessary to use a time series model suitable for nonstationary series. That is, the nonstationary sequence becomes a stationary sequence after d-order difference processing; then it is called the d-order single integer sequence. If a sequence can be transformed into a stationary sequence after several difference operations, then the sequence is called a homogeneous nonstationary sequence, and the number of previous differencing operations is called the homogeneous order. In the actual economic situation, the time series we get are usually nonstationary. In real life, most time series are nonstationary, showing trend or periodic characteristics, such as economic development data, electricity consumption, and passenger travel volume. The characteristic of ARIMA is that when dealing with nonstationary time series, it is first differentiated into a stationary time series, and then AR, MA, and ARMA model theories are used for modeling and analysis.  Figure 2 shows the structure topology of the ARIMA algorithm.

When predicting a stationary time series, the longer the prediction time is, the more the unknown information is represented. So a modified prediction is proposed; that is, as time develops, we can gradually get a real value, put this true value together with the previously known sequence value, refit the model, and then predict the value after again, and when the true value is obtained again, refit the model again, until the required predicted value until. If we use some methods to simplify the complex, convert the nonstationary to the stationary, and then use the ARMA model for fitting, we can solve the above problems. In the exponential smoothing model, the parameter smoothing coefficient can not only reflect the speed of response to changes in the time series, but also affect the ability to correct random errors. The magnitude of *a* determines the degree of influence of the new and old data on the forecast results.

### 2.3. GDP Forecast and Evaluation Method

**Figure 3:**
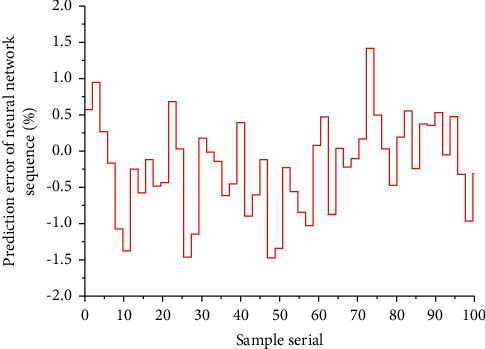
Prediction error ladder diagram of the BP neural network sequence.

According to the differences in GDP forecast goals and characteristics, people divide GDP forecasts into qualitative forecasts and quantitative forecasts. Qualitative forecasting is a subjective forecasting method, which is mainly used in the absence of historical data. In the event of statistical data, it is suitable for short, medium, and long-term forecasting. Quantitative forecasting is mainly based on a large amount of data through a mathematical model to explore the law between the data. The fitting and prediction of the sequence are the ultimate goal of analyzing the time series. After the model has passed the significance test, the sequence can be fitted. Combining several methods with an appropriate weight, combining geometric empirical mode decomposition and support vector regression method, the prediction index has good fit and accuracy. If the fitting effect is good, the model can be predicted; that is, according to the experience summarized in the forecasting work, when the data series shows a stable trend and no obvious fluctuations, it is more appropriate to choose between 0.1 and 0.3; when the data series shows a stable trend but there are obvious fluctuations, it should be between 0.3 and 0.5; when the data series has a clear trend and fluctuates, it is more reasonable to choose between 0.5 and 0.8.  Figure 3 shows the prediction error ladder diagram of the BP neural network sequence.

Since the prediction method changes with time, place, and environment, the predicted value will be different with the change of the prediction method, and there will be unavoidable errors between the actual value and the predicted value, so it needs to be formulated. A certain standard evaluates the feasibility and rationality of the prediction method as a basis for measuring the pros and cons of the model. When the fitted model passes the above two significance tests, it can only show that the model is at this level of significance, but it cannot be said that this model is optimal. There are generally two criteria for model optimization, AIC criteria and SBC criteria. Using a certain prediction method to predict it, and the resulting sequence of prediction results, the prediction error can be expressed as T. If there is a correlation between the time series values, it should test whether there is a stationary correlation between the series. This test is called the stationarity test of the series. Commonly used testing techniques include time series graph testing, autocorrelation function graph testing, and unit root testing to plot the time series data in a rectangular coordinate system and observe whether the broken line has periodicity and trend. If it is a stable time series, then the graph should fluctuate randomly around a certain value with a small amplitude. By observing the autocorrelation function graph of the sequence, if the autocorrelation coefficient shows a rapid decay state as the order *k* increases, then it can be determined that the sequence is stationary; conversely, if the decay is very slow, then the sequence is nonstationary.

### 2.4. Model Weight Factor Analysis

**Figure 4:**
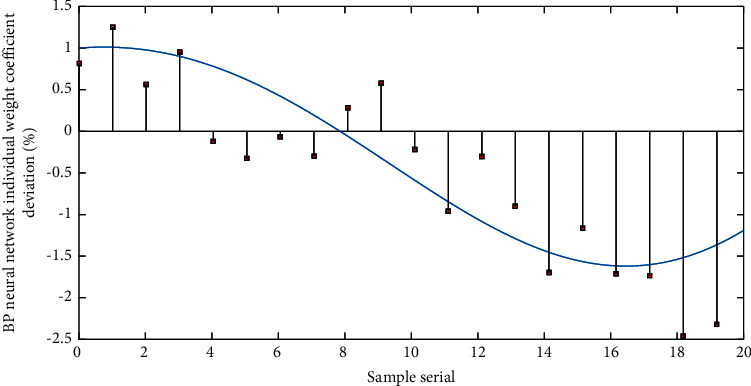
BP neural network individual weight coefficient deviation needle chart.

The key to GDP forecasting is what criteria are used to obtain the weight coefficients, so that the GDP forecast has a higher forecast accuracy, but no matter how the guidelines are formulated, the measurement standards are considered from the perspective of error, and the forecast error is inversely proportional to the weight distribution. The weight coefficient is determined by the principle. The nonoptimal positive weight combination is to use the above theory to solve the weight coefficient with a simple principle. It is obviously inferior to the objective function of the optimal positive weight combination in terms of calculation difficulty, but some nonoptimal positive weights are currently better and the calculation is simple. Sort the variance sums of the prediction errors of each single model, and assign smaller weight coefficients to the single model corresponding to the error variance and too large or too small, and the single-term model in the middle is assigned a larger weight coefficient.  Figure 4 shows the needle chart of the deviation of the individual weight coefficients of the BP neural network. The pros and cons of the prediction method can be reflected by the absolute value of the error. The maximum absolute value of the error is to be minimized based on the forecaster's needs, and the maximum absolute value of the GDP forecast error is minimized as much as possible, thereby improving the prediction accuracy.

For an observation time series, it is necessary to select the model that is most consistent with the actual development process from a variety of models, this is, the model identification process. That is to say, when predicting new unknown values, this model is not used to predict forever, but after predicting the first new value, the first value that is the furthest in time in the sequence is removed, and then the predicted value is added to the sequence, a new model is reconstructed, and so on, so as to improve the whitening degree of the gray space. Based on the tailing and truncation of the calculated autocorrelation function and partial correlation function, combined with the identification rules of the model, we preliminarily determine the model type. In terms of time series, for annual data, we use a data for modeling, and *b* data mainly is to compare forecasts. In addition, *c* data is predicted. For quarterly data, because the data is relatively small, *d* data is used to fit the model, and *e* data is used to determine whether the model is good or bad. It can be summarized as follows: when the autocorrelation function is truncated at step *q* and the partial correlation function is tailing, then the MA(*q*) model should be selected; when the partial correlation function is truncated at step *p*, and the autocorrelation function is tailing, then the AR(*p*) model is selected; if both the autocorrelation function and the partial correlation function show tailing, then the ARMA model is selected. Therefore, the combination coefficient method combining the least square method and the MAE weight coefficient method is proposed in the selection method of the combination model weight, and the difference in nature between different combination methods is compared. The best criterion function method uses a criterion function, which can not only examine the degree of fit to the original sequence, but also consider the number of unknown parameters in the model. If the minimum value is obtained under the established criterion function, the order of the model can be determined.

## 3. Results and Analysis

### 3.1. Empirical Analysis of the BP Neural Network

This article has elaborated on the relevant theories of time series and now constructs the GDP time series forecast model, using EViews software to establish the ARIMA model and the exponential smoothing model, respectively. This paper uses the Box-Jenkings model identification method to conduct preliminary identification of the model. The main idea of this method is to visually judge the truncation and tailing of the sequence by observing the autocorrelation function graph and partial correlation function graph of the sample and screening series five suitable model types. Before applying the dynamic regression model, it is necessary to test the stationarity of each series to avoid the appearance of false regression. The stationarity of the sequence can be observed through the sequence diagram of the sequence, but it has a strong subjective impression. In order to improve the accuracy of the test, we usually perform a unit root test on the sequence, which is the most widely used statistical test method. First, the correlation analysis of the variables was carried out using the Pearson correlation coefficient. According to the results obtained, it was found that the selected variables all showed a high degree of positive correlation with GDP, and the variables also showed a strong correlation.

**Figure 5:**
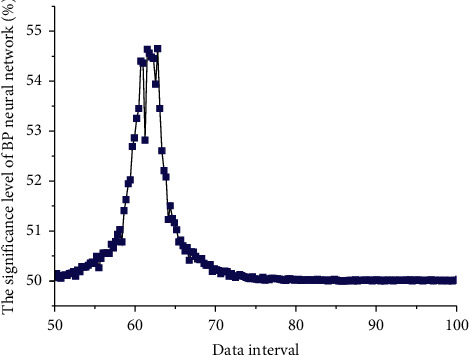
Distribution of saliency level curves of the BP neural network.

Figure 5 shows the distribution of each significance level curve of the BP neural network. From the figure, we can see that the GDP series has an obvious time trend, which is an exponential growth trend with time. It can be preliminarily judged that this is a nonstationary series. According to the ADF test, the test *t* statistic value is 1.493627, which is greater than the critical value at each significance level; that is, the null hypothesis cannot be rejected at the 1%, 5%, and 10% levels. Therefore, it can be judged that the original series is a nonstationary series. Since there are 6 independent variables in total, the input layer node is selected to be 6, the number of output layer nodes is 1, and the activation function is selected as a sigmoid type function. This is because it has good nonlinear mapping capabilities, and the hidden layer is selected as tan-sigmoid function; the output layer selects the linear purelin function. In order to eliminate the trend of the series and reduce the fluctuation of the series, we now take the logarithmic first difference of the original GDP series to get its series. According to the ADF test, the test *t* statistic value is −4.705133, which is less than the critical value under each significance level, that is, rejecting the null hypothesis, and it can be judged that the series is a stationary series at this time. Combining it can be seen that the sequence after the first difference of the logarithm of the original sequence is a stationary sequence, that is, *d* = 1. At this time, each parameter has a significant effect on the model, and the significance test of the parameter is passed. Then drawing the residual correlation diagram, it can be seen that there is no autocorrelation and heteroscedasticity in the residual, and it is normally distributed, and the model passes the test. Therefore, the established ARIMA(4, 1, 0) model meets the requirements.

### 3.2. Realization of GDP Prediction Model Simulation

**Figure 6:**
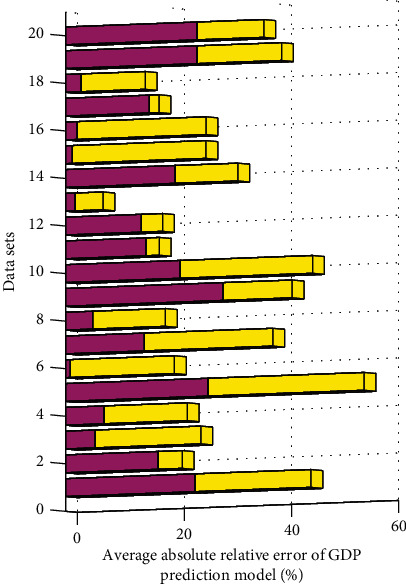
Histogram of the average absolute relative error of the GDP forecast model.

In this paper, statistical software SAS9.2 is used for ARIMA modeling of GDP total forecast; using the form of rolling window, the number of rolling windows is the number of forecast periods, and the data in the window is used to construct the ARIMA model. Sample 1 contains 18 windows for building models, and sample 2 contains 20 windows for building models. Due to space limitations, sample 1 and sample 2 only describe the modeling process of the first window in detail. The error fluctuation range of ARIMA prediction alone is large, and the error of BP prediction alone is relatively small. However, the combined prediction error is the smallest, and the obtained prediction data is closest to the actual value. The ARIMA model has certain adaptability to nonstationary demand. Compared with the ARIMA model, the BP neural network based on genetic algorithm has a greater improvement in the prediction accuracy. This is mainly due to the fact that the generation of data is random and uncertain, and most of the data contains white noise. For stationary non-white noise sequences, the stationarity can be predicted. Stationary non-white noise sequence refers to the correlation between the sequences; that is, there are rules to follow in the sequence. The ARIMA model is a linear model, which has shortcomings and defects in the analysis and prediction of this nonlinear behavior of change. According to the results of the simulation analysis of the calculation example, the combined model of the two methods can prove a great improvement compared with the single model.  Figure 6 shows the histogram of the average absolute relative error of the GDP forecast model.

According to the GDP sequence diagram, it can be roughly judged that GDP is growing in a weak exponential form. If the double exponential smoothing method is used to predict GDP, then the original sequence can be processed by logarithm, and then the sequence can be fitted with double exponential smoothing. After the model is calculated, it is transformed into an exponential curve trend form, and the predicted value of the GDP series can be obtained. In general, low-order numbers are selected when building the model, so let *p* = 1 and *q* = 1. From a long-term perspective, the autocorrelation graph shows two 4th-order truncation, and the partial autocorrelation graph shows tailing. That is, *P* = 1, *Q* = 1 or 2, and because the first-order 4-step difference is started, *d* = 1, *D* = 1, and *S* = 4. Note that this article uses EViews software to build an exponential smoothing model. The initial smoothing value is the system default value. The Alpha and Beta values let EViews automatically choose to minimize the error.

**Figure 7:**
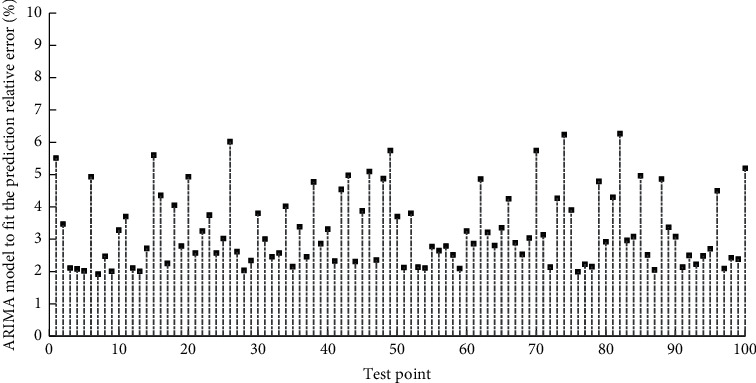
The matchstick chart of the relative error of the ARIMA model fitting prediction.

Figure 7 shows the matchstick chart of the relative error of the ARIMA model fitting prediction. It can be seen from the autocorrelation graph of the quadratic difference sequence that the autocorrelation coefficients fall within 2 times the standard deviation after a delay of 3 orders, and the speed of attenuation to zero is faster, and after a delay of 9 orders, it fluctuates around the zero value. Based on the above two judgment methods, it can be considered that the sequence after the second-order difference is stationary. After the fitted model is determined, the model needs to be tested in two aspects, one is the adaptability test of the model, and the other is the significance test of the parameters. Each neuron on the hidden layer and the output layer corresponds to an activation function and threshold. The neurons on each layer are connected to the neurons on the adjacent layer through weights. For a nonstationary time series, the nonstationarity is usually eliminated by difference operation and relevant information is extracted. But you cannot blindly use the difference operation multiple times, because every time a difference operation is performed, the time series will lose part of the information. When multiple differences of the time series result in too much information loss, the estimated model parameters are unreliable, which will reduce the value of the model. This phenomenon is called overdifferential. In order to avoid excessive differences, when a low-order difference operation can be used to obtain a stationary sequence, there is no need to use high-order differences. It can be seen from the above results that if only one year's data is predicted, according to the time series method, the forecast error of quarterly data is smaller than that of annual data. Therefore, if the data is sufficient, if the ARIMA model is used to fit the forecast data, the number predicted by the quarterly data is better than the data predicted by the annual data, because the quarterly data is more seasonal than the annual data. The adaptability test of the model is mainly to determine whether the model is valid by checking whether the residual sequence is a white noise sequence. The significance test of a parameter is to test whether the unknown parameter is significant to zero. If the independent variable corresponding to the parameter does not have a significant effect on the model, the variable should be considered to be eliminated.

### 3.3. Analysis of Experimental Results

The nonlinear component of the total GDP sequence is added to construct an ARIMA-BP mixed model. The nonlinear component in the input layer is the residual of the ARIMA model's first-order delay. The ARIMA-BP hybrid model will simultaneously model the linear component and the nonlinear component in the GDP total sequence. The structure of the BP neural network in the sample 1 construction of the ARIMA-BP mixed model is 5 × 6 × 1, and the structure in the sample 2 construction of the ARIMA-BP mixed model is 5 × 11 × 1. The model is trained to predict the daily closing price of the total GDP. The actual values and predicted values of BP neural network and ARIMA-BP mixed model can be found in the article. First, the data input_train and data output_train are used as the data of the training learning sample, and the data input_test and data output_test are used as the input and output data of the test sample, respectively. According to the parameter-related setting theory and multiple tests of the model, the appropriate model is selected according to the prediction effect. The model parameters are set as follows: the normalization function of the data is mapminmax; the number of hidden layers is set to 8; the number of iterations is net.trainParam.epochs = 1000; learning rate net.trainParam.lr = 0.1; training target net.trainParam.goal = 0.00001; other parameters are the system default settings.

**Figure 8:**
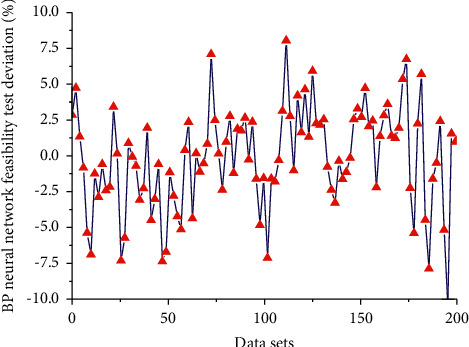
BP neural network feasibility test deviation line chart.

Figure 8 shows the BP neural network feasibility test deviation line chart. It is found from the result that when using all the data to test the feasibility of the model, the partial grade ratio does not fall within the tolerance interval, the data does not pass the feasibility test, and the gray model cannot be fitted. Cross-validation can evaluate the regression performance of LSSVM. It divides the data in the sample into multiple groups randomly. The training set trains LSSVM, and the validation set is used to test the prediction performance of LSSVM. After verification, a model that performs well in the sample can be used for out-of-sample predictions. RMSE measures the absolute error, and MAPE measures the relative error. RMSE and MAPE measure the accuracy of prediction from two different perspectives, and the two do not have similarities. For example, the RMSE index of the prediction effect of the A model is smaller than that of the B model, but the MAPE index of the prediction effect of the A model may be greater than that of the B model. Therefore, it is feasible to choose RMSE and MAPE to evaluate the predictive effects of different models in this paper. The smaller the two indicators, the closer the actual value to the predicted value, and the higher the prediction accuracy. When the level ratio test fails, one solution is to add a constant to the original sequence until the new sequence passes the level ratio test. The amount of change in the weight and threshold is equal to the amount of change in the previous moment. By increasing the momentum term, the network not only has a faster convergence speed, but also can effectively avoid the occurrence of local minimum problems in network training. Experiments show that if all the sequences are tested, only the constant is increased to 65000 or even more. The test can only be passed when it is large, and the predicted sequence, as shown in the text, can only roughly reflect a rising trend of GDP year by year, but the actual effect of predicting the future is very poor.

Table 2 shows the sequence-level comparison test of the neural network. It can be found that the process of ACF and PACF attenuation to zero presents a tailing feature. The tailing order is the first order and the fourth order, which fits the ARMA(4, 1) model. Taking into account the process of first-order difference, the ARIMA(4, 1, 1) model was finally constructed on the basis of logarithms. The equation passed the significance test, but some coefficients failed the significance test. The reason is considered and compared, and the sparse coefficient model ARIMA((1, 3), 1, 1) is finally constructed, and the *R* language operation result is obtained. The prediction interval is trumpet-shaped, indicating that the prediction error is increasing with the growth of the prediction period. In addition to the significance test of the parameters, the adequacy of the model must also be verified; that is, the significance of the model is tested. If a model is significantly effective, it has extracted sufficient information from the time series, and the fitted residual series are no longer relevant. If the residual sequence is white noise, it indicates that the model is significantly effective; on the contrary, when the residual sequence is correlated, it indicates that the model does not sufficiently extract the correlation relationship in the time sequence. The logarithm operation was performed during the stationarity test of the original sequence at the beginning. Therefore, the predicted value obtained through the program on the basis of the logarithm needs to be exponentially operated to return to the actual required value. According to the formula for determining the weight of the correlation coefficient at the *k* time, the improved correlation coefficient matrix is obtained.

## 4. Conclusion

GDP forecast mainly includes two important aspects, one is the choice of a single model, and the other is the combination of models. In the choice of a one-way model, this article chooses the differential autoregressive moving average model (referred to as the ARIMA model), and BP neural network model and exponential curve model are studied. In terms of combination methods, this article combines weighted combination and error correction combination on the single model. After processing the sequence and identification, we use time series analysis theory 67ARIMA (*p*, *d*, *q*) model to predict GDP, and finally the graph is intuitively fitted well, and compared with the real value of the GDP prediction result, the relative error is small, and the result is more reasonable. The predicted value of the time series is fitted to the GDP forecast, and the results are compared and analyzed with the ARMA model. First, an ARIMA model and an exponential curve regression model are established, and then MAPE and least squares are finally used to predict the GDP using a comprehensive weighted GDP prediction model based on MAPE and least squares. The simulation results show that the comprehensive weight is compared with a single weight. It is found that the forecasting effect of the combined model is better than that of a single model, and the combined weight coefficient method proposed in this paper is better than the combined model composed of other weight coefficient methods in the prediction error. In terms of the accuracy of GDP forecasting, the accuracy of GDP forecasting can be improved; in terms of error correction combination, this paper establishes gray adaptive filtering and ARIMA-BP according to the principle of complementary advantages and commonality of the model and establishes two error correction GDP forecasting models, which are verified by examples. The results show that the neural network has strong nonlinear mapping ability and good robustness, can identify and distinguish noisy samples, and is also a better GDP prediction model.

## Figures and Tables

**Table 1:** Time series factor composition.

Factor index	Network sequence	Coefficient	Significance level
1	AR	1.71	0.58
2	MA	2.43	0.43
3	AR (*D*)	1.63	0.39
4	MA (*Q*)	1.56	0.54

**Table 2:** Sequence-level comparison test of the neural network.

Level number	Correlation coefficient	Weight	Error rate/%
1	0.97	0.32	9.17
2	0.86	0.34	5.43
3	0.88	0.21	6.77
4	0.91	0.13	3.54

## Data Availability

The data used to support the findings of this study are available from the corresponding author upon request.

## References

[1] Du Y. Application and analysis of forecasting stock price index based on combination of ARIMA model and bp neural network.

[2] Jiang F., Yang X., Li S. (2018). Comparison of forecasting India’s energy demand using an MGM, ARIMA model, MGM-ARIMA model, and BP neural network model. *Sustainability*.

[3] Shi X. (2020). Tourism culture and demand forecasting based on BP neural network mining algorithms. *Personal and Ubiquitous Computing*.

[4] Yu Z., Qin L., Chen Y., Parmar M. D. (2020). Stock price forecasting based on LLE-BP neural network model. *Physica A: Statistical Mechanics and Its Applications*.

[5] Zhao X., Han M., Ding L., Calin A. C. (2018). Forecasting carbon dioxide emissions based on a hybrid of mixed data sampling regression model and back propagation neural network in the USA. *Environmental Science and Pollution Research*.

[6] Weng Y., Wang X., Hua J., Wang H., Kang M., Wang F.-Y. (2019). Forecasting horticultural products price using ARIMA model and neural network based on a large-scale data set collected by web crawler. *IEEE Transactions on Computational Social Systems*.

[7] Li Z., Li Y. (2020). A comparative study on the prediction of the BP artificial neural network model and the ARIMA model in the incidence of AIDS. *BMC Medical Informatics and Decision Making*.

[8] Qi D. (2020). Study on the export of BP neural network model to China based on seasonal adjustment. *Advances in Intelligent Systems and Computing*.

[9] Ji S., Yu H., Guo Y. (2016). Research on sales forecasting based on ARIMA and BP neural network combined model. *Intelligent Information Processing*.

[10] Zhang Y., Fu Y., Li G. (2020). Research on container throughput forecast based on ARIMA-BP neural network. *Journal of Physics: Conference Series*.

[11] Gao Y., Qu C., Zhang K. (2016). A hybrid method based on singular spectrum analysis, firefly algorithm, and BP neural network for short-term wind speed forecasting. *Energies*.

[12] Qin J., Tao Z., Huang S. (2021). Stock price forecast based on ARIMA model and BP neural network model. *Big Data, Artificial Intelligence and Internet of Things Engineering*.

[13] Yang H., Li X., Qiang W., Zhao Y., Zhang W., Tang C. (2021). A network traffic forecasting method based on SA optimized ARIMA-BP neural network. *Computer Networks*.

[14] Wang L., Zhan L., Li R. (2019). Prediction of the energy demand trend in middle africa-A comparison of MGM, MECM, ARIMA and BP models. *Sustainability*.

[15] Wang D., Luo H., Grunder O., Lin Y., Guo H. (2017). Multi-step ahead electricity price forecasting using a hybrid model based on two-layer decomposition technique and BP neural network optimized by firefly algorithm. *Applied Energy*.

[16] Liu Y., Tian Y., Chen M. (2017). Research on the prediction of carbon emission based on the chaos theory and neural network. *International Journal Bioautomation*.

[17] Zhang L., Wang F., Xu B., Chi W., Wang Q., Sun T. (2018). Prediction of stock prices based on LM-BP neural network and the estimation of overfitting point by RDCI. *Neural Computing & Applications*.

[18] Yang Y., Chen Y., Wang Y., Li C., Li L. (2016). Modelling a combined method based on ANFIS and neural network improved by DE algorithm: a case study for short-term electricity demand forecasting. *Applied Soft Computing*.

[19] Wang D., Luo H., Grunder O., Lin Y. (2017). Multi-step ahead wind speed forecasting using an improved wavelet neural network combining variational mode decomposition and phase space reconstruction. *Renewable Energy*.

[20] Li W., Wang Y. (2021). Dynamic evaluation of logistics enterprise competitiveness based on machine learning and improved neural network. *Journal of Ambient Intelligence and Humanized Computing*.

[21] Shao-Jiang L., Jia-Ying C., Zhi-Xue L. (2018). A EMD-BP integrated model to forecast tourist number and applied to Jiuzhaigou. *Journal of Intelligent and Fuzzy Systems*.

[22] Cao J., Wang J. (2020). Exploration of stock index change prediction model based on the combination of principal component analysis and artificial neural network. *Soft Computing*.

[23] Wang D., Liu Y., Wu Z., Fu H., Shi Y., Guo H. (2018). Scenario analysis of natural gas consumption in China based on wavelet neural network optimized by particle swarm optimization algorithm. *Energies*.

[24] Bozkurt Ö. Ö., Biricik G., Tayşi Z. C. (2017). Artificial neural network and SARIMA based models for power load forecasting in Turkish electricity market. *PLoS one*.

[25] Gao Y., Cao Y., Jiang Z. (2019). Investment forecast of power network infrastructure project based on BP neural network. *IOP Conference Series: Earth and Environmental Science*.

[26] Al-Maqaleh B. M., Al-Mansoub A. A., Al-Mansoub A. A., Al-Badani F. N. (2016). Forecasting using artificial neural network and statistics models. *International Journal of Education and Management Engineering*.

[27] Sutthichaimethee P., Wahab H. A. (2021). A forecasting model in managing future scenarios to achieve the sustainable development goals of Thailand’s environmental law: enriching the path analysis-varima-ovi model. *International Journal of Energy Economics and Policy*.

[28] Sun W., Wang Y. (2018). Short-term wind speed forecasting based on fast ensemble empirical mode decomposition, phase space reconstruction, sample entropy and improved back-propagation neural network. *Energy Conversion and Management*.

